# The porcine virome and xenotransplantation

**DOI:** 10.1186/s12985-017-0836-z

**Published:** 2017-09-06

**Authors:** Joachim Denner

**Affiliations:** Robert Koch Fellow, Robert Koch Institute, Nordufer, 20 Berlin, Germany

**Keywords:** Porcine viruses, Virome, Xenotransplantation, Porcine endogenous retroviruses, Porcine cytomegalovirus, Porcine circoviruses, Hepatitis E virus

## Abstract

The composition of the porcine virome includes viruses that infect pig cells, ancient virus-derived elements including endogenous retroviruses inserted in the pig chromosomes, and bacteriophages that infect a broad array of bacteria that inhabit pigs. Viruses infecting pigs, among them viruses also infecting human cells, as well as porcine endogenous retroviruses (PERVs) are of importance when evaluating the virus safety of xenotransplantation. Bacteriophages associated with bacteria mainly in the gut are not relevant in this context. Xenotransplantation using pig cells, tissues or organs is under development in order to alleviate the shortage of human transplants. Here for the first time published data describing the viromes in different pigs and their relevance for the virus safety of xenotransplantation is analysed. In conclusion, the analysis of the porcine virome has resulted in numerous new viruses being described, although their impact on xenotransplantation is unclear. Most importantly, viruses with known or suspected zoonotic potential were often not detected by next generation sequencing, but were revealed by more sensitive methods.

## Background

Xenotransplantation is being developed to overcome the shortage of human tissues and organs needed to treat organ failure by allotransplantation. Pigs are the preferred species to be used as donor animals for a number of reasons including the size of the animals, their similar physiology, and the ease with which they can be genetically modified and cloned.

Although there are still some hurdles that have to be overcome, such as immunological rejection, physiological incompatibility and risk of transmission of porcine microorganisms, recent achievements in breeding genetically modified pigs, in the development of new immunosuppressive regimens and in approaches to safety suggest that xenotransplantation may soon be introduced in the clinic (for review see [1–3]).

In the past, only a few porcine viruses with known or suspected ability to infect human cells or to accelerate transplant rejection have been analysed within the context of xenotransplantation. Next generation sequencing or deep sequencing techniques and metagenomic analyses now give the opportunity to analyse the entire virome of pigs and its impact on xenotransplantation. These studies on the pig virome are, like investigations into the virome of humans and other species, only at their very early stages [4]. The development of metagenomics has revolutionised virus discovery, leading to the identification of many new viruses. These studies will generate an enormous amount of data concerning the prevalence of porcine viruses although these will be difficult to interpret, especially with regard to the virus safety of xenotransplantation.

## Xenotransplantation and public health

By March 3, 2017, 118,265 waiting list candidates were listed at UNOS (United network for organ sharing), among them 98,149 for kidney transplantations. However, in 2016 only 19,061 kidney transplantations were performed in the US, indicating the increasing gap between supply and demand, and the figures for heart transplantations were 3191 versus 3994 (https://www.unos.org/). Xenotransplantation could help to overcome this shortage. Another field where xenotransplantation may have an enormous impact on public health is the treatment of diabetes. In 2012, 29.1 million Americans, or 9.3% of the population, had diabetes, among them approximately 1.25 million children and adults with type 1 diabetes (http://www.diabetes.org/diabetes-basics/statistics/). Although the treatment of diabetes type 1 with insulin was quite successful in the past using pig insulin and only recently recombinant human insulin, complications were often observed, mainly due to insufficient compliance of the patients leading to limb amputation and blindness. The total cost of diagnosed diabetes in the United States in 2012 was 245 billion dollars, most of which was spent on the treatment of complications while expenditure for insulin was relatively low. A biologically regulated system based on porcine islet cells would help avoid these complications [5]. Xenotransplantation therefore has the potential to have an enormous impact on public health.

## The porcine virome

As described in the abstract, the pig virome is the total amount of viruses in and on the pig body and includes also the endogenous retroviruses. Initial attempts to analyse the porcine virome were undertaken using samples of the gut or faeces. These studies were started to better understand the reasons for neonatal porcine diarrhoea, a common problem worldwide that contributes to morbidity and mortality among piglets [6]. Although investigations of the porcine intestinal virome are so far limited [7–9], samples from the distal jejunum of 19 healthy pigs in a Swedish conventional herd yielded eight different viral families (Table 1). The most common were picornaviruses, followed by circoviruses [10]. Viral infections were already observed 24 to 48 h after birth, indicating infection immediately post-partum, or alternatively, transplacental infection. Similar results were obtained when Chinese pigs were analysed [7] where, in addition to the viruses identified in Sweden, the coronaviruses transmissible gastroenteritis virus (TGEV) and porcine epidemic diarrhoea virus (PEDV) were found (Tables 2 and 3). In this study, the virus prevalence rates revealed that all samples were co-infected with at least two different viruses and that, notably, co-infection with 11 different viruses was identified in one sample [7]. Detailed analyses of German sows showed that the pig faecal virome is highly variable and its general composition is mainly dependent on the age of the pigs [11]. Kobuviruses were predominantly detected in 12 day-old piglets, bocaviruses and others in 54 day-old piglets and circoviruses in the sows [11, 12].

**Table 1:** Main virus families of the pig virome [10]

Virus family	Characterisation^a^	Prevalence in healthy pigs (%, *n* = 19)	Prevalence in Swedish diarrhoeic pigs (%, *n* = 10)
*Adenoviridae*	dsDNA	16	0
*Anelloviridae*	ssDNA	5	10
*Astroviridae*	ssRNA	5	0
*Caliciviridae*	ssRNA	5	0
*Circoviridae*	ssDNA	42	10
*Parvoviridae*	ssDNA	11	0
*Picornaviridae*	ssRNA	53	40
*Reoviridae*	dsRNA	11	20

^a^ *ss* single stranded, *ds* double stranded

**Table 2:** Main viruses in the faeces of healthy Chinese pigs [7]

Virus	%	Virus family
Porcine bocavirus	7	*Parvoviridae*
Porcine epidemic diarrhea virus (PEDV)	7	*Coronaviridae*
Sapovirus	17	*Caliciviridae*
Sapelovirus	17	*Picornaviridae*
Torovirus	7	*Coronaviridae*
Posavirus-1	17	*Picornaviridae*
Porcine astrovirus	72	*Astroviridae*
Porcine enterovirus-9	86	*Picornaviridae*
Kobuvirus	90	*Picornaviridae*

**Table 3:** Main viruses in the faeces of diarrhoeic Chinese pigs [7]

Virus	%	Virus family
Porcine bocavirus-2	59	*Parvoviridae*
Porcine bocavirus-4	18	*Parvoviridae*
Torque teno sus virus-2 (TTSuV-2)	7	*Anelloviridae*
Porcine epidemic diarrhea virus (PEDV)	70	*Coronaviridae*
Porcine stool associated circular virus (PoSCV)	7	*?*
Po-circo-like	11	*?*
Sapovirus	33	*Caliciviridae*
Sapelovirus	48	*Picornaviridae*
Torovirus	33	*Coronaviridae*
Posavirus-1	40	*Picornaviridae*
Porcine astrovirus	74	*Astroviridae*
Coronavirus	7	*Coronaviridae*
Porcine enterovirus-9	85	*Picornaviridae*
Picobirnavirus (PBV)	15	*Picobirnaviridae*
Kobuvirus	44	*Picornaviridae*

Viral metagenomics analysis in healthy and pigs suffering from the postweaning multisystemic wasting syndrome (PMWS) showed that pigs have a considerable viral flora consisting mainly of small single-stranded and circular DNA viruses [13]. Although many viruses were also detected in the healthy pigs, the number of porcine circovirus 2 (PCV2) had increased considerably in the PMWS pigs.

When pig mucus (the raw material for production of heparin) was investigated, parvoviruses (76%, within this group bocaviruses represent 80%), circoviruses (16%) and picornaviruses (2.5%) were mainly found [14]. Viruses known to be transmissible to humans such as influenza virus, hepatitis E virus (HEV) and encephalomyocarditis virus (ECMV) [15, 16] were not found.

When the virome of feral swine (wild boars) in the US was analysed, a total of 16 different viruses were identified [17]. Mainly single stranded DNA viruses were detected including *Circoviridae, Anelloviridae* and *Parvovirinae.* Torque teno virus (*Anelloviridae*) was the most commonly detected virus (73%), PCV2 was identified in 13% of the samples. Only four RNA viruses were identified: kobuvirus, sapelovirus, pestivirus and an orthopneumovirus [17]. This is in contrast to previous studies of the faecal virome which were dominated by RNA viruses [6, 7]. Feral swine in Texas were also commonly infected with influenza A virus and the H1N1 2009 virus [18], representing a reservoir for domestic pigs and zoonotic infection [18–20]. Meanwhile it was shown that influenza virus transmission from human to swine is far more frequent than swine-to-human zoonosis [21]. This implies that the staff working in facilities producing pigs for xenotransplantation should be screened for influenza virus.

It is important to underline, that cells and organs for xenotransplantation will be derived from pigs kept under specified pathogen free (SPF) or defined pathogen free (DPF) conditions. SPF means that the herds are free from a list of specified pathogens. SPF piglets are usually produced by Caesarean delivery and reared under sterile conditions. During this time they may be given a probiotic flora and sterilised colostrum supplement. They are then removed from the isolator and placed in a very clean room under hygienic conditions (http://www.thepigsite.com/pighealth/article/29/by-pigs/). This means that the virome of the donor pigs will be well-defined. The knowledge of the virome of feral and farmed animals will be useful in understanding the prevalence of certain viruses and for the development of detection methods and elimination programs.

Since PERVs cannot be eliminated this way, they represent a special risk for xenotransplantation (see below).

For comparison it may be interesting to give a short summary about research on the human virome. The human virome was determined in the context of faecal transplantation against Clostridium difficile infection [22]. When analysing longitudinal variation in the human gut virome, loss and acquisition of viral types was found uncommon [23]. When the blood virome in 8000 humans was investigated, 94 different viruses were found, among them 19 DNA viruses [24]. These viruses were found in 42% of the individuals with a copy number of two to millions per 100,000 cells. Herpesviruses (altogether 41.6%) and anelloviruses (8.91%) were the most common viruses (Table 4). Among the viruses were numerous potentially pathogenic viruses such as Merkel cell polyomavirus (MCPV), human immunodeficiency virus (HIV), human T cell lymphotropic virus (HTLV), papillomaviruses and others.

**Table 4:** Detected human viruses in blood DNA of 8240 humans [24]

Virus	Abbreviation	Percentage of individuals
Human herpesvirus 7	HHV-7	20.38
Human herpesvirus 4 (Epstein Barr virus)	HHV-4 (EBV)	14.45
Anellovirus	TTV & TLMV^a^	8.91
Human herpesvirus 6A	HHV-6A	4.80
Human herpesvirus 6B	HHV-6B	1.47
Merkel cell polyomavirus	MCPV	0.59
Human herpesvirus 5 (Human cytomegalovirus)	HHV-5 (HCMV)	0.35
Human T-lymphotropic virus 1/2	HTLV-1/2	0.27
Human papillomavirus	HPV	0.19
Human herpesvirus 1 (Herpes simplex virus)	HHV-1 (HSV)	0.12
Human parvovirus B19		0.12
Human adenovirus		0.11
Human immunodeficiency virus 1/2	HIV-1/2	0.06
Human herpesvirus 8 (Kaposi sarcoma herpesvirus)	HHV-8 (KSHV)	0.04
Human polyomavirus		0.04
Hepatitis C virus	HCV	0.01

^a^ *TTV* Torque teno virus, *TLMV* TTV-like minivirus

It is interesting that samples from sick individuals have yielded viruses more frequently than samples from healthy controls [25, 26] and that infection with one virus may significantly change the composition of the virome. When the influence of a HIV-1 infection on the virome in humans was analysed, significant changes were observed when a clinically validated cutoff of less than 200 CD4^+^ cells/ml was used. Significantly more *Adenoviridae* and *Anelloviridae* sequences were detected in HIV-positive subjects with CD4^+^ lower than 200 cells/ml when compared with HIV-negative and HIV-positive subjects with CD4^+^ greater 200 [27], indicating that expansion of both viruses is associated with HIV induced immunosuppression.

## Risk posed by porcine viruses

In addition to the possible transmission of porcine bacteria and fungi, which may be eliminated using antibiotics and antimycotics, transmission of viruses through xenotransplantation may lead to disease in the recipient, i.e. zoonoses [28]. For most porcine viruses there are neither antivirals nor vaccines available. However, it is still unclear whether porcine viruses can actually infect humans and cause zoonoses. In contrast to human pathogens that are well adapted to humans, porcine microorganisms are not. For only a few porcine viruses a zoonotic potential has been described, for example for HEV, genotype 3. HEV induces a chronic infection in immunosuppressed patients and severe disease in patients with a pre-existing liver failure (for review see [29, 30]). Of special interest is the porcine cytomegalovirus (PCMV) which may be indirectly pathogenic without infecting cells of the host. In preclinical trials of transplanting pig kidneys into cynomolgus monkeys and baboons, the presence of PCMV led to early transplant failure (for review see [31]). Since there is still no evidence for PCMV infection of non-human primate as well as human cells, the organ failure was possibly due to cytokines produced in response to viral antigens [31].

Porcine lymphotropic herpesvirus (PLHV) 1, 2, and 3 are common porcine herpesviruses, but their prevalence and importance for xenotransplantation is not well understood [32, 33]. PLHV may be transmitted by pre-partum cross-placental vertical transfer or post-partum horizontal transmission, although the former is relatively rare. Between 26% and 88% of animals in different herds in Germany, Ireland, France, Spain and the United States are infected with one of the PLHV variants [34–37] and, unlike PCMV, early weaning cannot eradicate PHLV [38].

Porcine circoviruses (PCV), the smallest viruses replicating autonomously in mammalian cells, are also widely distributed (for review see [39]). There are two types, porcine circovirus 1 (PCV1), which is apathogenic in pigs, and PCV2, which causes a severe multifactorial disease (PCV2 disease, PCVD, formerly post-weaning multisystemic wasting syndrome, PMWS). Although the presence of PCV2 is necessary for the disease, additional factors are also required and the severity of the disease depends on the status of the immune system and genetic predisposition. There is no serological evidence indicating transmission of PCV2 to humans and indeed, the case of a human rotavirus vaccine being contaminated with PCV demonstrated that PCV was not transmitted to the vaccinated humans [40, 41]. Infection experiments with human cell lines showed persistence of PCV1 in most cell lines without causing any visible changes, but cytopathogenic effects in PCV2-transfected cells [42]. Most importantly, the infection appeared to be non-productive [42, 43].

It is interesting to note that porcine viruses with a known or suspected zoonotic potential such as the herpesviruses PCMV and PLHV were not detected when viromes were analysed in faeces and blood [7–10] whereas PCV (mainly PCV2) was commonly found [7–10]. Presumably, the frequency of the herpesviruses is so low that their DNA cannot be detected by next generation sequencing. Indeed, only low numbers of herpesvirus sequences were found in German sows [11, 12] and in swine intestinal mucus, the raw material for the manufacture of heparin [14]. Similarly, next generation sequencing of human samples failed to identify low-level viruses that are revealed using viral particle enrichment or PCR amplification [44, 45]. The most effective approach was serological profiling, detecting virus-specific antibodies in human sera using a synthetic human virome [46]. Testing 569 human donors across four continents with this method revealed antibodies to an average of 10 human viral species per person and 84 species in at least two individuals [46].

Whereas the zoonotic potential of many pig viruses is still unknown, some viruses are well characterised zoonotic viruses. One example is the hepatitis E virus (HEV) which can be easily transmitted by consumption of undercooked pork or by contact to pigs and which can induce disease and chronic infection in immunosuppressed individuals and in people with liver failure (for review see [30]). A second example is the paramyxovirus Nipah virus, which was transmitted from pigs to humans mainly working in slaughterhouses in Malaysia and Singapore [47, 48]. The virus induced severe infections of the respiratory tract and encephalitis in humans, 48% of the infected died [47, 48]. The Menangle virus was first described in a piggery in Australia experiencing a high number of stillbirths and deformities during farrowing. Two workers at the piggery got infected and had a serious flu-like illness [49]. Nipah virus and Menagle virus had their origin in fruit bats [50]. Rabies is considered a rare disease in pigs, however an infection with the rabies virus is invariably fatal in all species including the human [49, 51]. Another rhabdoviruses, the vesicular stomatitis virus (VSV) can infect insects, cattle, horses and pigs and is zoonotic, inducing flu-like illness in infected humans [52]. The Eastern equine encephalomyelitis virus (EEEV) and the Japanese encephalomyelitis virus (JEV) are two arboviruses infecting pigs. The transmission cycle of the JEV involves pigs as virus amplifiers and mosquitoes as vectors for transferring the virus between amplifying hosts and to dead-end hosts, i.e. humans, horses and cattle [53]. In pigs, it can cause abortion and stillbirths. In an outbreak of EEEV infection in swine, 280 of 350 pigs died. Histopathologic changes in the brain, multifocal necrosis and inflammation in the myocardium were seen. Growth retardation was noticed in surviving pigs [54]. Pigs can be protected from EEEV infection by a vaccine [55]. Domestic swine in the Philippines have been discovered to host Reston ebolavirus (REBOV) [56]. Although REBOV is the only member of Filoviridae that has not been associated with disease in humans, its emergence in the human food chain is of concern.

## Porcine endogenous retroviruses

Whereas the viruses discussed above may be eliminated by selection, treatment, vaccination, Caesarean delivery, early weaning and embryo transfer, this is impossible in the case of porcine endogenous retroviruses (PERVs). These viruses are the result of infections with retroviruses in the distant past. Retroviruses possess enzymes able to transcribe the viral RNA genome into DNA copies and to integrate them into the cellular genome to form proviruses [57]. If oocytes or sperm cells happen to become infected, these proviruses will be present in all cells of the developing progeny and will be inherited in the same way as all other genes. Endogenous retroviruses have been found in all mammals including humans [57]. However, in contrast to the human endogenous retroviruses which are mostly defective and do not produce infectious particles, many retroviruses in other species remain active. Gamma- and betaretroviruses have been found integrated into the genome of pigs [58, 59] and sequencing of the entire pig genome (*Sus scrofa*) revealed 212 PERV insertions in the genome [60]. The gammaretroviruses include PERV-A and PERV-B, which are integrated into the genome of all pigs, and PERV-C, found in many (but not all) pigs. PERVs are produced as infectious virus particles and may infect human cells [61–63]. In addition, recombinants between PERV-A and PERV-C have been found that are highly replication-competent (for review see [64]). PERVs, like most other retroviruses, may theoretically induce tumours and/or immunodeficiency, but their zoonotic potential remains unknown and no PERV transmission was observed in pig-to-non-human primate preclinical trials and initial clinical trials [63, 65–67]. Recently all PERVs in the genome of an immortalised PK15 cell line (62 copies) and in primary fibroblasts (25 copies) were inactivated using CRISPR/Cas specific for the highly conserved *pol* gene of PERV [68, 69]. After a somatic cell nuclear transfer live and healthy pigs with inactivated PERVs could be generated [69]. This approach reduces the risk posed by PERVs to zero.

## Conclusion

Next generation sequencing and metagenomic analyses allow the entire virome of pigs to be characterised and will result in the detection of many new porcine viruses. However, the impact of these viruses on xenotransplantation is unclear. Until now, porcine viruses with known or suspected zoonotic potential, although often detected by other methods, have been rarely detected by next generation sequencing and their detection appears to require other, more sensitive methods. Furthermore, it remains unclear whether there are other potentially zoonotic viruses not detected by next generation sequencing and not yet investigated using more sensitive methods.

## Abbreviations

ds: double stranded
EBV: Epstein Barr virus
ECMV: Encephalomyocarditis virus
H1N1 2009 virus: haemagglutinin 1 and neuraminidase 1 2009 influenza virus
HCMV: Human cytomegalovirus
HCV: Hepatitis C virus
HEV: Hepatitis E virus
HHV-1: human herpesvirus 1
HHV-4: Human herpesvirus 4
HHV-5: Human herpesvirus 5
HHV-6A: Human herpesvirus 6A
HHV-6B: Human herpesvirus 6B
HHV-7: Human herpesvirus 7
HHV-8: Human herpesvirus 8
HIV-1/2: Human immunodeficiency virus 1/2
HPV: Human papillomavirus
HSV: Herpes simplex virus
HTLV-1/2: Human T-lymphotropic virus 1/2
KSHV: Kaposi sarcoma herpesvirus
MCPV: Merkel cell polyomavirus
PBV: Picobirnavirus
PCV1: Porcine circovirus 1
PCV2: Porcine circovirus 2
PCVD: PCV2 disease
PERV: Porcine endogenous retroviruses
PLHV: Porcine lymphotropic herpesvirus
PMWS: Post-weaning multisystemic wasting syndrome
PoSCV: Porcine stool associated circular virus
pPEDV: porcine epidemic diarrhoea virus
ss: single stranded
TGEV: Transmissible gastroenteritis virus
TLMV: TTV-like minivirus
TTSuV-2: Torque teno sus virus-2
TTV: Torque teno virus
